# Postoperative Day-28 Neutrophil-to-Lymphocyte Ratio as a Predictor of Early Mortality After Lung Transplantation

**DOI:** 10.3390/diagnostics16081170

**Published:** 2026-04-15

**Authors:** Hyeon Kyeong Bae, Shihwan Chang, Ala Woo, Chanho Lee, Mindong Sung, Kyung Soo Chung, Song Yee Kim, Jin Gu Lee, Moo Suk Park, Young Sam Kim, Su Hwan Lee, Ah Young Leem

**Affiliations:** 1 Division of Pulmonary and Critical Care Medicine, Department of Internal Medicine, Severance Hospital, Yonsei University College of Medicine, Seoul 03722, Republic of Korea; 2 Division of Pulmonary, Allergy and Critical Care Medicine, Chung-Ang University Gwangmyeong Hospital, Gwangmyeong 14353, Republic of Korea; 3 Department of Thoracic and Cardiovascular Surgery, Severance Hospital, Yonsei University College of Medicine, Seoul 03722, Republic of Korea

**Keywords:** lung transplantation, neutrophil–lymphocyte ratio, neutrophils, lymphocytes, mortality

## Abstract

**Background/Objectives**: Neutrophil-to-lymphocyte ratio (NLR) may predict outcomes after organ transplantation. This study evaluated the peri-transplant prognostic value of NLR in lung transplantation (LTx). **Methods**: This retrospective study included 282 LTx recipients (2012–2020). NLR measured on PODs 1, 3, 7, and 28 predicted 6-month mortality. Generalized estimating equations analyzed serial trends. Multivariable regression and ROC analysis identified predictors for a composite model, assessing discrimination and calibration. **Results**: Among 282 recipients (mean age, 54.2 years; male, 65.2%; idiopathic pulmonary fibrosis, 54.3%), 24.1% died within 6 months, most commonly from infection. Median NLR increased sharply after LTx (pre-LTx, 5.4; POD 1, 23.1; POD 3, 31.2), then decreased (POD 7, 18.8; POD 28, 8.7). Non-survivors had significantly higher preoperative and postoperative NLRs, particularly on POD 28. POD 28 NLR independently predicted 6-month mortality (multivariable analysis: OR, 1.05 per unit; 95% CI, 1.02–1.07; *p* < 0.001), alongside age and donor lung PaO_2_/FiO_2_ (P/F) ratio. Notably, a composite model combining these variables demonstrated significantly superior discrimination (area under the curve [AUC], 0.742; *p* = 0.001) compared to the NLR-only model (AUC, 0.698; *p* < 0.05). GEE demonstrated significantly steeper post-transplant NLR decline among survivors than non-survivors after adjusting for age (*p* = 0.02). Patients with NLR > 9.20 at POD 28 (area under the curve, 0.698; 95% CI, 0.615–0.782; sensitivity, 71.4%; specificity, 59.8%)—showed significantly lower survival on Kaplan–Meier analysis (*p* < 0.001, log-rank). **Conclusions**: Persistent NLR elevation on POD 28 independently predicts early mortality post-LTx and may support routine post-transplant risk stratification.

## 1. Introduction

Since the first successful human lung transplantation (LTx) by James Hardy in 1963 [1], various immunosuppressive drugs and surgical techniques have been developed to ensure long-term survival. In the 21st century, with pharmaceutical developments and improvements in techniques and allocation protocols, LTx is an established treatment option for patients with end-stage lung disease. However, while other solid-organ transplants have achieved survival times lasting over a decade, the median survival time for LTx procedures has stagnated at 5.63 years [2]. Repetitive tissue injury, repair, and chronic inflammation may be major contributors to this stagnation [2,3].

Several causes of mortality have been reported in patients undergoing LTx. Infection remains a persistent risk across all post-transplant phases; primary graft dysfunction is the leading cause of death within 30 days after LTx, and chronic lung allograft dysfunction is a major cause of mortality after 1 year [4]. These outcomes are closely linked to inflammatory responses: end-stage lung disease induces pre-transplant inflammation [5], transplant surgery itself triggers inflammatory and immune responses, and lifelong immune modulation is required to maintain graft function [6]. Several biomarkers have been proposed to identify inflammation status, including C-reactive protein, hypoalbuminemia, neutrophil count, leukocyte differentials, composite indices such as neutrophil-to-lymphocyte ratio (NLR), platelet-to-lymphocyte ratio, systemic immune-inflammation index, as well as pro-inflammatory cytokines such as interleukin (IL)-1α, IL-1β, IL-6, and tumour necrosis factor alpha. Among these, NLR received particular attention because it can be easily obtained through a common complete blood count.

NLR is increasingly recognised as a prognostic marker in critically ill and surgical patients [7], and an elevated NLR has been found in diverse conditions, including infection [8], various types of malignancy [9,10,11], acute coronary syndrome [12], and even COVID-19 infection [13]. Furthermore, recent studies have revealed that elevated perioperative NLR is associated with graft failure in organ transplants [14,15]. Based on these studies, we aimed to investigate whether baseline NLR is associated with 6-month mortality and trends in NLR during the early posttransplant period.

## 2. Materials and Methods

### 2.1. Study Design and Population

This single-centre retrospective cohort study reviewed the data of 292 consecutive LTx recipients between October 2012 and August 2020 at a tertiary hospital in South Korea. The exclusion criteria were the following: (1) age < 16 years old, (2) patients who previously received a LTx and were receiving a re-transplantation, (3) those who received simultaneous multiple-organ transplantation, such as heart-lung or kidney-lung transplantation.

All patients received cardiopulmonary bypass or extracorporeal membrane oxygenation (ECMO) support during transplantation surgery. High-dose corticosteroids (250 mg of methylprednisolone intraoperatively, followed by 0.5 mg/kg/day for 3 days) were administered to all patients. Postoperatively, all recipients received a triple immunosuppressive regimen consisting of corticosteroids, calcineurin inhibitors, and mycophenolate mofetil. In addition, ganciclovir and itraconazole or voriconazole were administered to all transplant recipients for 6 months after surgery. Trimethoprim/sulfamethoxazole for the prevention of Pneumocystis jirovecii pneumonia was prescribed for life.

### 2.2. Data Collection and Definition

We collected patient data, including demographics, laboratory results, comorbidities, level of care required during admission (non-intensive vs. intensive care), whether bridged ECMO was applied, and clinical outcome at 6 months (death vs. no death) from the electronic medical records of the hospital. The primary indication for lung transplantation was defined as the underlying lung disease that directly led to end-stage respiratory failure. For statistical analysis, each patient was assigned to a single primary diagnosis category; in cases where multiple pulmonary conditions coexisted, the dominant etiology for transplantation was selected. Systemic comorbidities, such as hypertension and diabetes, were recorded as separate clinical variables. NLR was calculated by dividing the neutrophil count by the lymphocyte count from the differential white cell count. Donor medical information, such as age, sex, and height, was obtained from the Korean Network Organ Sharing (KONOS). The KONOS status is a classification system used to prioritize candidates based on medical urgency: Status 0 for patients requiring mechanical ventilation or ECMO; Status 1 and 2 for those with varying degrees of oxygen requirements; and Status 3 for those with end-stage lung disease but not requiring oxygen at rest. The KONOS score (ranging from 0 to 11) is a comprehensive value determined by this status, waiting duration, and the patient’s age to ensure fair organ allocation.

### 2.3. Outcomes

The primary endpoint was the association between NLR and 6-month mortality, and the secondary endpoints were changes in NLR over time and related clinical implications. NLR was measured at baseline (pre-LTx) and at postoperative day (POD) 1, 3, 7, and 28. The patients were categorised into two groups according to their 6-month survival status. Multivariate logistic regression analyses were performed to identify independent predictors of mortality.

### 2.4. Ethical Statement

The study protocol was approved by the institutional review board of Severance Hospital (approval no. 4-2025-1456). This study adhered to the principles of the Declaration of Helsinki (2000) and the Declaration of Istanbul (2008).

### 2.5. Statistical Analysis

Continuous variables are described as mean ± standard deviation for normally distributed data and as median with interquartile range (IQR) for non-normally distributed data. Student’s *t*-tests were used to compare parametric continuous characteristics; for non-normally distributed variables, the Mann–Whitney U test was used instead. Categorical variables are expressed as numbers and percentages and compared using Pearson’s chi-square analysis.

Univariate and multivariate logistic regression analyses were used to investigate the factors affecting 6-month mortality. The optimal cutoff value for NLR was determined using receiver operating characteristic (ROC) curve analysis. To evaluate the incremental prognostic value of routine clinical variables, a composite multivariable prediction model was constructed. The discrimination of the composite model and the NLR-only model was compared using the DeLong test, and model calibration was assessed using the Hosmer–Lemeshow goodness-of-fit test. Additionally, internal validation via a bootstrapping procedure with 1000 resamples was performed to assess the stability of the predictive model and address potential overfitting. Kaplan–Meier survival analysis was used to estimate survival curves, and the log-rank test was applied to compare them.

Serial changes in NLR findings at different time points were compared between the two groups using generalised estimating equations (GEE) to estimate the parameters of a generalised linear model with possible unmeasured correlations after LTx. IBM SPSS Statistics (v. 26.0; IBM Corp., Armonk, NY, USA) was used for all statistical analyses.

## 3. Results

### 3.1. Baseline Patient Characteristics and Comparisons Relative to 6-Month Mortality

Overall, 282 patients were included in this study (Table 1). The mean age was 54.2 years, and 184 (65.2%) participants were male. The most common underlying disease was idiopathic pulmonary fibrosis (IPF) (n = 153, 54.3%). Before LTx, 182 (64.5%) patients were admitted to the intensive care unit (ICU), and 90 (31.9%) received ECMO as a bridge to transplantation. The median KONOS score was 1. The mean PaO_2_/FiO_2_ (P/F) ratio of donor lungs was 452.0 ± 95.3, the donor/recipient total lung capacity (D/R TLC) was 107.7 ± 17.9, and the median donor age was 45 years. The median NLR was 5.4 preoperatively, increased to 23.1 at POD 1 and 31.2 at POD 3, and then decreased to 18.8 at POD 7 and 8.7 at POD 28.

Among the 282 included patients, 68 (24.1%) died within 6 months of LTx. The most common cause of death was infection (n = 34, 50%), followed by cardiac (n = 11, 16.2%) and other complications comprising renal or hepatic failure (n = 7, 10.3%), thrombotic microangiopathy (n = 13, 19.1%), and unknown causes (n = 3).

There were significant differences between the survivor and non-survivor groups in age, preoperative NLR, POD 1 NLR, POD 7 NLR, POD 28 NLR, albumin level, days in the ICU before transplantation, and donor lung P/F ratio (Table 1). The mean age of 6-month survivors was 53.3 years, while that of non-survivors was 57.2 years (*p* = 0.01). The median preoperative NLR was higher in non-survivors than in survivors (6.9 vs. 5.2, *p* = 0.01). Similarly, postoperative NLR values were significantly different between the two groups on POD 1 (17.1 vs. 24.1, *p* = 0.019), POD 7 (22.9 vs. 18.1, *p* = 0.03), and POD 28 (15.7 vs. 7.9, *p* < 0.05). There were no differences in underlying disease, ICU admission, ICU stay pre-transplantation, ECMO application, or ECMO support duration between the two groups.

### 3.2. Factors Associated with Mortality in Univariate and Multivariate Analysis

In the univariate analysis (Table 2), age, preoperative NLR, POD 28 NLR, baseline albumin, days of ECMO before LTx, and donor lung P/F ratio were significantly different between the two 6-month mortality groups.

Multivariate analysis was performed using variables that showed significant differences in the univariate analysis. In the multivariate analysis (Table 3), age, POD 28 NLR, and donor lung P/F ratio were independently associated with 6-month mortality. Notably, POD 28 NLR was an independent risk factor for 6-month mortality, with an odds ratio of 1.05 (95% confidence interval [CI]: 1.02–1.07, *p* < 0.001).

### 3.3. Serial Trend of NLR and Related Clinical Impact

**Figure 1:**
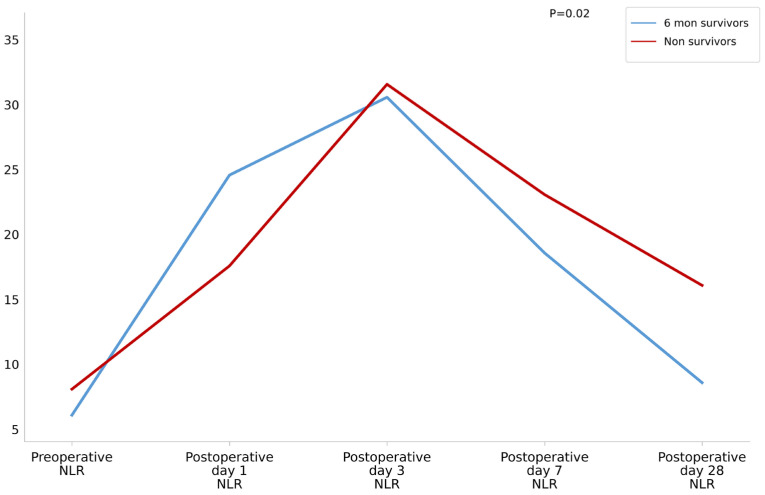
Changes in neutrophil-to-lymphocyte ratio according to 6-month mortality.

In both groups, NLR tended to increase and then decrease after LTx. Moreover, the survivor group showed a significantly sharp decline in NLR compared to the non-survivor group after adjusting for age (*p* = 0.02) (Figure 1).

**Figure 2:**
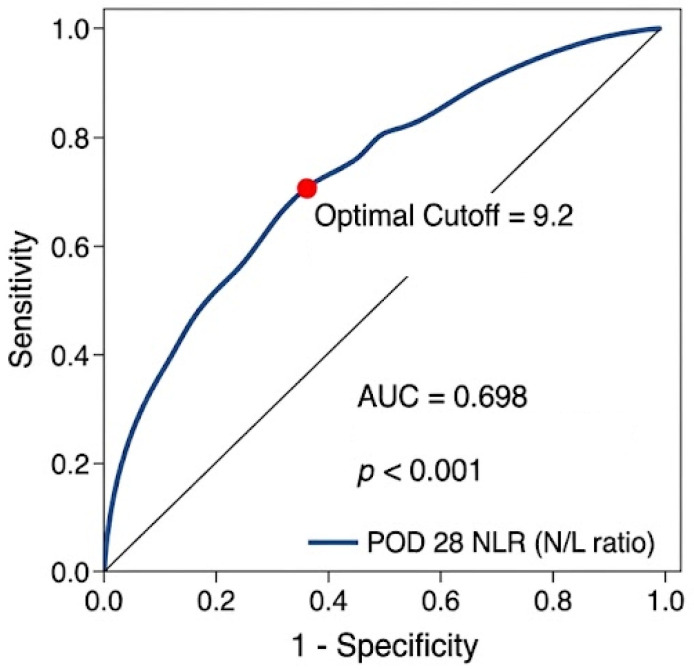
Receiver operating characteristic (ROC) curve of the neutrophil-to-lymphocyte ratio (NLR) at postoperative day (POD) 28 for predicting 6-month mortality.

Because significant differences in NLR trends and absolute values were observed at POD 28, we determined the optimal cutoff for POD 28 NLR associated with mortality using ROC curve analysis (Figure 2). An NLR cutoff of 9.2 at POD 28 was identified (area under the curve, 0.698; 95% CI, 0.615–0.782; sensitivity, 71.4%; specificity, 59.8%). To assess the stability of this cutoff and address potential overfitting, internal validation via a bootstrapping procedure with 1000 resamples was performed. This analysis yielded an optimism-corrected AUC of 0.698, with an optimism-corrected sensitivity and specificity of 71.4% and 59.8%, respectively. These metrics were nearly identical to the apparent performance, indicating minimal overfitting and confirming the robustness of the model. Baseline demographic variables, such as age and sex, did not differ between groups stratified by this cutoff (Table 4). Baseline demographic variables, such as age and sex, did not differ between groups stratified by this cutoff (Table 4).

**Figure 3:**
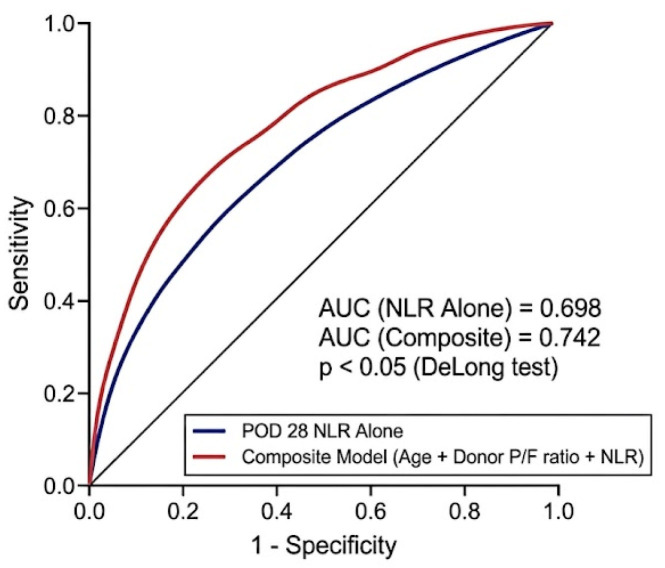
Comparison of receiver operating characteristic (ROC) curves between the single and composite prediction models for 6-month mortality.

Furthermore, to determine whether routine clinical variables add meaningful prognostic value, we constructed a composite prediction model incorporating patient age, donor P/F ratio, and POD 28 NLR. This composite model demonstrated superior discrimination, yielding an AUC of 0.742 (95% CI, 0.658–0.826), which was significantly higher than that of the NLR-only model (*p* < 0.05 by DeLong test; Figure 3). Additionally, both models showed adequate calibration with no significant lack of fit (Hosmer–Lemeshow test, *p* = 0.684 for the composite model and *p* = 0.421 for the NLR-only model).

**Figure 4:**
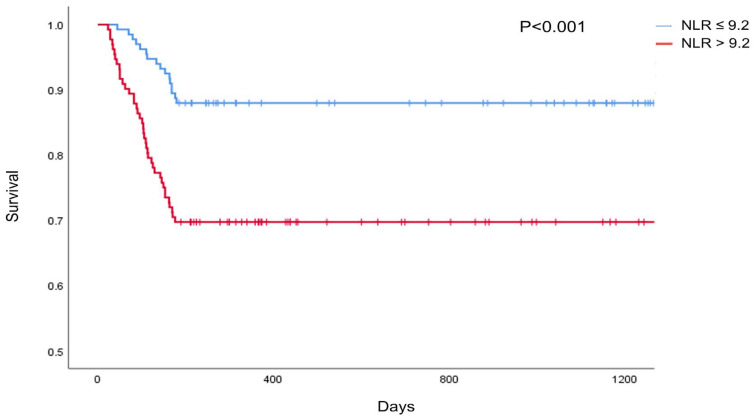
Kaplan–Meier survival analysis comparing survival days based on neutrophil-to-lymphocyte ratio of 9.2.

However, NLR values before and after LTx were consistently significantly lower in the group with POD 28 NLR ≤ 9.2. Mortality outcomes also differed significantly: in patients with POD 28 NLR > 9.2, the 6-month mortality rate was 31.2% (39/125), compared with 11.7% (17/145) in those with POD 28 NLR ≤ 9.2 (*p* < 0.001). Kaplan–Meier analysis confirmed significantly lower survival in the high NLR group (log-rank *p* < 0.001) (Figure 4). Furthermore, in terms of lung function, the POD 28 NLR ≤ 9.2 group showed that forced vital capacity at 3, 6, and 12 months was significantly higher than that in the POD 28 NLR > 9.2 group.

## 4. Discussion

Although some studies have previously investigated the association between NLR and clinical outcomes such as graft failure and mortality in solid organ transplant recipients [15,16,17,18], this is the first study to assess serial postoperative NLR following LTx and demonstrate its association with mortality. In this study, we found that elevated preoperative and postoperative NLRs are associated with poor prognosis after LTx; in particular, NLR at POD 28 was independently associated with increased 6-month mortality.

A cutoff value of 9.2 at POD 28 effectively stratified patients by risk, with a higher NLR being associated with increased mortality and poorer pulmonary function at follow-up. Our findings are consistent with those of previous studies showing that an elevated preoperative NLR is predictive of long-term outcomes after solid organ transplantation, including graft failure and mortality [15,16,17,18]. Krishnan et al. reported that among 95 patients with LTx, those with elevated preoperative NLR (>4) demonstrated a higher incidence of graft failure and increased 3-year mortality [15]. Similarly, elevated NLR has been associated with graft failure in kidney and heart transplantation [14,17]. Previous studies have primarily focused on preoperative NLR values, whereas our study established the assessment of serial NLR values obtained preoperatively and postoperatively, as well as POD 28 NLR thresholds that contribute to a more refined risk stratification in the immediate postoperative phase following LTx.

Interestingly, our data revealed that the survivor group exhibited a higher NLR during the hyper-acute postoperative phase (POD 0 to 3) compared to non-survivors. This paradoxical pattern may be explained by the profound physiological response to surgical trauma and high-dose induction corticosteroids. High-dose steroids rapidly induce neutrophil demargination and lymphocyte sequestration, typically causing an NLR peak within the first few days. A robust early NLR elevation in survivors might therefore reflect a physiologically intact response to these massive exogenous stimuli. Conversely, the critical prognostic differentiator appears to be the trajectory of NLR resolution. Survivors demonstrated a rapid decline in NLR, reflecting the successful resolution of acute systemic inflammation, whereas non-survivors showed persistent or secondary elevations from POD 7 onwards. This suggests that the failure to resolve early inflammation is a stronger determinant of early mortality than the magnitude of the initial inflammatory hit.

The mechanistic link between persistently elevated NLR at POD 28 and increased 6-month mortality likely stems from a profound and sustained imbalance between innate and adaptive immunity. Regarding the specific immune compartments, persistent neutrophilia reflects ongoing activation of the innate immune system, actively contributing to allograft damage. Neutrophils infiltrating the allograft release reactive oxygen species (ROS), proteolytic enzymes, and neutrophil extracellular traps (NETs), which exacerbate epithelial injury and drive fibrotic remodeling [19]. Conversely, concurrent lymphopenia impairs adaptive immune responses and severely depletes the immune reserve, significantly increasing susceptibility to fatal opportunistic infections [20,21,22].

Importantly, an elevated NLR at POD 28 serves as a composite biomarker reflecting the final common pathway of unresolved systemic stress. Rather than pointing to a single etiology, it indicates unmitigated allograft injury driven by unresolved primary graft dysfunction (PGD), occult infections, or subclinical acute rejection [23,24,25]. Furthermore, the intensity of the immunosuppressive regimen plays a critical role in mediating this association. High-dose corticosteroids induce robust neutrophil demargination and lymphocyte apoptosis, while aggressive maintenance therapy profoundly suppresses lymphocyte proliferation [26,27]. Therefore, a persistently elevated NLR may reflect not only an ongoing inflammatory milieu but also a state of ‘over-immunosuppression.’ These findings highlight NLR’s potential as a comprehensive prognostic tool indicative of infection severity, immune-mediated graft injury, and pharmacological stress. In addition, NLR offers clinical utility because of its simplicity, cost-effectiveness, and accessibility through routine laboratory testing.

Nonetheless, this study has some limitations. The retrospective, single-centre design of this study may limit the generalizability of our findings. Additionally, owing to the retrospective nature of the study, detailed time-varying data regarding cumulative corticosteroid doses and the precise documentation of active infection status precisely at POD 28 were not fully adjusted for in the multivariable analysis. Because NLR is highly sensitive to exogenous high-dose steroids and occult infections, these unmeasured variables may act as both confounders and clinical mediators. While an elevated NLR inherently captures the net burden of these critical post-transplant events, the inability to perfectly isolate the intrinsic prognostic value of NLR from these specific treatment and infection effects remains a limitation that warrants future prospective investigation. Furthermore, the observational design of this study precludes any inference of causality. Finally, we acknowledge the modest discrimination (AUC = 0.698) and low specificity (59.8%) of the NLR-only model. Relying solely on an elevated NLR risks false-positive classifications, potentially leading to unnecessary invasive procedures, antimicrobial overuse, and increased healthcare costs. Therefore, rather than a standalone diagnostic trigger, NLR should serve as an early ‘red flag’ for closer monitoring, ideally integrated with other prognostic variables as shown in our composite model. Future studies should validate these findings, adopting a prospective design, in diverse patient populations and evaluate whether NLR-guided interventions can lead to improved clinical outcomes.

## 5. Conclusions

In conclusion, an elevated POD 28 NLR is an independent predictor of 6-month mortality in LTx recipients. Furthermore, integrating NLR with routine clinical parameters into a composite model significantly enhances prognostic accuracy. Therefore, routine NLR monitoring, when interpreted within a broader clinical context, may serve as a simple and valuable tool for post-transplant risk stratification and management.

## Figures and Tables

**Table 1:** Baseline patient characteristics according to 6-month survival.

Variables	All Patients (n = 282)	Survivor (n = 214)	Non Survivor (n = 68)	*p*-Value
Age (years)	54.2 ± 12.3	53.3 ± 12.5	57.2 ± 11.1	0.010
Male sex	184 (65.2)	139 (65.0)	45 (66.2)	0.854
Preoperative NLR (IQR)	5.4 (3.3–11.0)	5.2 (3.0–10.2)	6.9 (3.9–21.8)	0.010
POD 1 NLR	23.1 (14.3–31.9)	24.1 (14.6–34.6)	17.1 (11.7–30.0)	0.019
POD 3 NLR	31.2 (20.8–46.9)	31.1 (20.6–49.7)	31.6 (22.3–41.5)	0.546
POD 7 NLR	18.8 (12.9–27.6)	18.1 (12.4–25.9)	22.9 (14.6–34.6)	0.030
POD 28 NLR (IQR) (n = 270)	8.7 (2.0–15.6)	7.9 (4.5–12.3)	15.7 (7.5–33.1)	<0.001
Albumin (g/dL)	3.3 (2.9–3.9)	3.3 (2.9–4.0)	3.1 (2.6–3.7)	0.007
BMI (kg/m^2^)	20.9 ± 4.1	20.7 ± 4.2	21.6 ± 4.1	0.102
Underlying diseases, n (%)				
IPF	153 (54.3)	115 (53.7)	38 (55.9)	0.757
CTD-ILD	48 (17.0)	35 (16.4)	13 (19.1)	0.597
Bronchiectasis	19 (6.7)	16 (7.5)	3 (4.4)	0.380
Lymphangioleiomyomatosis	5 (1.8)	5 (2.3)	0.0 (0)	0.203
COPD	9 (3.2)	7 (3.3)	2 (2.9)	0.893
Bronchiolitis obliterans	20 (7.1)	16 (7.5)	4 (5.9)	0.655
Others	29 (10.3)	20 (9.3)	8 (11.8)	0.273
Hypertension	67 (23.8)	52 (24.3)	15 (22.1)	0.706
DM	77 (27.3)	57 (26.6)	20 (24.1)	0.654
ICU Pre-Transplant	182 (64.5)	133 (62.1)	49 (72.1)	0.137
Days of ICU prior to transplant	0.0 (0.0–12.0)	0.0 (0.0–11.0)	4.5 (0.0–14.0)	0.029
ECMO Pre-Transplant	90 (31.9)	62 (29.0)	28 (41.2)	0.060
Days of ECMO pre-transplant	0.0 (0.0–5.3)	0.0 (0.0–3.0)	0.0 (0.0–9.8)	0.063
Single lung transplant	11 (3.9)	7 (3.3)	4 (5.9)	0.333
KONOS status	1.0 (1.0–2.0)	1.0 (1.0–2.0)	1.0 (0.25–2.0)	0.932
Donor age	45.0 (33.0–52.0)	45.0 (31.0–52.8)	45.0 (7.8–52.0)	0.932
P/F ratio (n = 275)	452.0 ± 95.3	461.4 ± 91.7	421.0 ± 101.1	0.003
D/R TLC (n = 273)	107.7 ± 17.9	106.9 ± 17.3	110.1 ± 19.6	0.209

Data are expressed as mean ± standard deviation or median (interquartile range). Data are presented as column percentages. NLR, neutrophil–lymphocyte ratio; POD, postoperative day; BMI, body mass index; IPF, idiopathic pulmonary fibrosis; CTD-ILD, connective tissue disease-associated interstitial lung disease; COPD, chronic obstructive pulmonary disease; DM, diabetes mellitus; ICU, intensive care unit; ECMO, extracorporeal membrane oxygenation; KONOS, Korean Network Organ Sharing; P/F, PaO_2_/FiO_2_ (ratio); D/R TLC, donor/recipient total lung capacity.

**Table 2:** Univariate logistic regression analysis for factors associated with 6-month mortality.

Variables	Univariate Analysis		
	aOR	95% Cl	*p*-Value
Age (years)	1.03	1.00–1.06	0.023
Male sex	1.06	0.60–1.88	0.854
Preoperative NLR	1.05	1.02–1.07	0.010
POD 1 NLR	0.99	0.97–1.01	0.178
POD 3 NLR	1.00	1.00–1.01	0.695
POD 7 NLR	1.01	1.00–1.03	0.106
POD 28NLR	1.05	1.03–1.08	<0.001
Albumin (g/dL)	0.55	0.36–0.85	0.007
BMI (kg/m^2^)	1.06	0.99–1.13	0.103
ICU pre-transplant	0.64	0.35–1.16	0.139
Days of ICU prior to transplant	1.02	1.00–1.04	0.141
ECMO pre-transplant	0.58	0.33–1.03	0.062
Days of ECMO pre-transplant	1.03	1.00–1.06	0.044
Single lung transplant	0.54	0.15–1.91	0.339
KONOS status (IQR)	1.07	0.80–1.42	0.645
Donor age (IQR)	1.02	1.00–1.04	0.188
P/F ratio	1.00	0.99–1.00	0.004
D/R TLC	1.01	1.00–1.02	0.210

NLR, neutrophil-to-lymphocyte ratio; POD, postoperative day; BMI, body mass index; ECMO, extracorporeal membrane oxygenation; ICU, intensive care unit; KONOS, Korean Network Organ Sharing; P/F, PaO_2_/FiO_2_ (ratio); D/R TLC, donor/recipient total lung capacity.

**Table 3:** Multivariate logistic regression analysis for factors associated with 6-month mortality.

Variables	Multivariate Analysis		
	aOR	95% Cl	*p*-Value
Age (years)	1.04	1.00–1.08	0.045
Male sex	1.42	0.63–3.19	0.394
Preoperative NLR	1.03	0.99–1.06	0.167
POD 28 NLR	1.05	1.02–1.07	<0.001
Albumin (g/dL)	0.55	0.29–1.05	0.068
BMI (kg/m^2^)	1.045	0.96–1.14	0.329
ICU pre-transplant	1.13	0.46–2.75	0.794
Days of ICU before transplant	0.99	0.94–1.03	0.508
ECMO pre-transplant	0.33	0.09–1.17	0.085
Days of ECMO pre-transplant	1.04	0.98–1.12	0.213
KONOS status (IQR)	1.14	0.79–1.64	0.495
P/F ratio	0.99	0.99–1.00	0.001
D/R TLC	1.01	0.99–1.03	0.633

NLR, neutrophil-to-lymphocyte ratio; POD, postoperative day; BMI, body mass index; ICU, intensive care unit; ECMO, extracorporeal membrane oxygenation; KONOS, Korean Network Organ Sharing; P/F, PaO_2_/FiO_2_ (ratio); D/R TLC, donor/recipient total lung capacity.

**Table 4:** Baseline patient characteristics according to NLR cut-off value of 9.2.

Variables	NLR ≤ 9.2 (n = 145)	NLR > 9.2 (n = 125)	*p*-Value
Age (years)	57 (46–63)	58.0 (50.5–63.0)	0.499
Male sex	96 (66.2)	79 (63.2)	0.606
Preoperative NLR	4.8 (2.7–8.2)	6.7 (3.9–15.6)	0.001
POD 1 NLR	20.7 (13.9–29.4)	25.2 (15.1–38.1)	0.025
POD 3 NLR	27.8 (19.6–40.7)	35.5 (22.9–53.1)	<0.001
POD 7 NLR	15.4 (10.2–21.3)	22.8 (16.6–34.4)	0.003
POD 28 NLR	5.2 (3.4–7.3)	16.4 (11.7–25.5)	<0.001
Albumin (g/dL)	3.3 (2.9–4.0)	3.3 (2.8–3.9)	0.155
BMI (kg/m^2^)	20.6 ± 4.1	21.1 ± 4.3	0.377
Underlying diseases, n (%)			
IPF	89 (61.4)	58 (46.4)	0.014
CTD-ILD	21 (14.5)	25 (20.0)	0.229
Bronchiectasis	9 (50.0)	9 (7.2)	0.744
Lymphangioleiomyomatosis	4 (2.8)	1 (0.8)	0.234
COPD	1 (0.7)	8 (6.4)	0.009
Bronchiolitis obliterans	10 (6.9)	10 (8.0)	0.730
Others	11 (7.6)	15 (12.0)	0.220
Hypertension	28 (19.3)	37 (29.6)	0.049
DM	36 (24.8)	40 (32.0)	0.191
ICU Pre-Transplant	91 (62.8)	81 (64.8)	0.728
Days of ICU prior to transplant	0.0 (0.0–14.0)	0.0 (0.0–8.0)	0.236
ECMO Pre-Transplant	42 (29.0)	42 (33.6)	0.412
Days of ECMO pre-transplant	0.0 (0.0–7.0)	0.0 (0.0–3.5)	0.903
Single lung transplant	3 (2.1)	8 (6.4)	0.073
KONOS status	1.1 (1.0–2.0)	1.0 (0.5–2.0)	0.515
Donor age	46.0 (31.0–52.0)	45.0 (34.0–52.8)	0.841
P/F ratio (n = 275)	451.8 ± 94.0	452.2 ± 95.8	0.972
D/R TLC (n = 273)	108.2 ± 18.7	106.8 ± 17.1	0.526
Pulmonary function test of 6-month survivors, n (%)			
3-month FVC (n = 115 vs. n = 50)	61 (49–71)	53 (41.8–61)	0.002
3-month FEV1 (n = 115 vs. n = 50)	69 (57–84)	62.5 (47.8–73)	0.170
3-month DLCO (n = 92 vs. n = 43)	65.5 (54.3–78.8)	59 (46–72)	0.156
6-month FVC (n = 115 vs. n = 51)	67 (52–77)	56 (41–65)	0.007
6-month FEV1 (n = 115 vs. n = 51)	72 (58–88)	64 (49–74)	0.019
6-month DLCO (n = 103 vs. n = 43)	65 (52–78)	62 (44–76)	0.578
12-month FVC (n = 97 vs. n = 48)	70 (56.5–80.5)	62 (53–68.8)	0.001
12-month FEV1 (n = 97 vs. n = 48)	75 (62–89.5)	68 (55.5–78.5)	0.156
12-month DLCO (n = 88 vs. n = 41)	56.3 (50.7–80.8)	59 (47.5–71)	0.067

Data are expressed as mean ± standard deviation or median (interquartile range). Data are presented as column percentage. DLCO, diffusing capacity of the lungs for carbon monoxide; FVC, forced vital capacity; NLR, neutrophil–lymphocyte ratio; POD, postoperative day; BMI, body mass index; IPF, idiopathic pulmonary fibrosis; CTD-ILD, connective tissue disease-associated interstitial lung disease; COPD, chronic obstructive pulmonary disease; DM, diabetes mellitus; ICU, intensive care unit; FEV1, forced respiratory volume in 1 s; ECMO, extracorporeal membrane oxygenation; KONOS, Korean Network Organ Sharing; P/F, PaO_2_/FiO_2_ (ratio); D/R TLC, donor/recipient total lung capacity.

## Data Availability

The data that support the findings of this study are available from the corresponding author upon reasonable request.

## Abbreviations

The following abbreviations are used in this manuscript:

NLR	Neutrophil-to-lymphocyte ratio
LTx	lung transplantation
POD	postoperative day
GEE	generalised estimating equations
IQR	interquartile range
ROC	receiver operating characteristic
BMI	body mass index
IPF	idiopathic pulmonary fibrosis
CTD-ILD	connective tissue disease-associated interstitial lung disease
COPD	chronic obstructive pulmonary disease
DM	diabetes mellitus
ICU	intensive care unit
ECMO	extracorporeal membrane oxygenation
P/F	PaO_2_/FiO_2_ (ratio)
D/R TLC	donor/recipient total lung capacity
